# Teacher motivational strategies in Saudi university EFL writing classes: a qualitative study

**DOI:** 10.3389/fpsyg.2025.1483456

**Published:** 2025-08-13

**Authors:** Muhammad M. M. Abdel Latif, Talal Musaed Alghizzi, Tahani Munahi Alshahrani

**Affiliations:** ^1^Faculty of Graduate Studies of Education, Cairo University, Giza, Egypt; ^2^College of Languages and Translation, Imam Mohammad Ibn Saud Islamic University (IMSIU), Riyadh, Saudi Arabia

**Keywords:** writing motivation, writing de-motivation, motivational strategies, writing teacher, L2 writing, Saudi universities

## Abstract

Empirically, motivating students to write is an issue yet to be given due research attention. Some previous relevant works have suggested guidelines for motivating writing students, but the studies qualitatively exploring the realities of writing teachers’ use of motivational strategies remain scant. In this study, we investigated Saudi university teachers’ perceptions of their students’ English writing de-motivation symptoms (i.e., signs or indicators) and causes of lack of motivation to write, and the ways they motivate students to write and participate in classroom activities. We explored these issues through using interviews with 33 teachers (17 males and 16 females) who had English writing instruction experiences at five Saudi universities. The 33 teachers identified seven main symptoms of students’ writing de-motivation (procrastinating assignment submission, engaging rarely in classroom activities, showing writing apprehension, copying others’ writing, skipping classes, perceiving writing value negatively, and experiencing writing block), and they referred to five causes of it (students’ poor language and writing ability, uninteresting topics, ineffective teaching, previous poor experiences, and the cognitive nature of writing). The teachers also reported using eight main motivational strategies in their English writing classes. For these teachers, class size is a very influential factor in their use of motivational strategies. The results generally suggest that writing motivation is yet to be given more attention in Saudi university English writing classes. The study provides the following recommendations: fostering teacher motivation literacy, activating the use of motivational strategies in writing classes, and minimizing class size.

## 1 Introduction

Second language (L2) students’ motivation plays an important role in their language acquisition and learning. Therefore, due attention should be paid to nurturing L2 students’ motivation. The task of motivating students to write is a much more complicated one than motivating them to learn a language (Abdel Latif, 2021). In classes covering multiple L2 areas, it is normal to find more than one teacher instructing students, and thus assuming the responsibility of motivating them to learn the target language. In writing classes, only the writing teacher is responsible for motivating students to write. What makes motivating students to write a more challenging task for the teacher is the fact that writing is the most cognitive of all the language skills as it requires much more time and deeper mental processes.

Despite its importance, the issue of how teachers motivate their students to write has been rarely researched. There have been a few relevant empirical studies on the realities of using motivation strategies (Cheung, 2018; Lee and Lin, 2022; Mali, 2017; Rosina, 2017; Saranraj et al., 2014). Meanwhile, other published relevant works have only provided guidelines for motivational strategies in writing classes (e.g., Abdel Latif, 2019a, 2019b, 2021; Bruning and Horn, 2000; Reeves, 1997; Troia et al., 2012; Walker, 2003). Thus, such obvious scarcity requires conducting further research on teachers’ use of motivational strategies in L2 writing classes. Specifically, we need to understand writing teachers’ perceptions of their students’ de-motivation symptoms and sources, how they try to motivate them to write, and the factors influencing teachers’ use of motivational strategies in writing classes. Understanding these issues could help in identifying what writing teachers need to make their instruction more motivating and how to help writing students avoid de-motivation symptoms. The present study attempted to tackle this under-explored research area by examining Saudi university English-as-a-foreign-language (EFL) teachers’ perceptions of their students’ writing de-motivation and its causes, the motivational strategies they use in writing classes, and the role of students’ writing competence and classroom size as potential factors influencing their use of motivational strategies.

## 2 Writing motivation and teacher motivational strategies

Writing motivation is a multifaceted construct encompassing a number of sub-constructs. It can be generally defined as “learners’ liking or disliking of writing situations and perceived value of writing, the situational feelings they experience while writing and the way they regulate them, the beliefs about their writing ability and skills, and their desired goals for learning to write” (Abdel Latif, 2021, p. 3). In light of this taxonomy, writing motivation constructs can be classified into the following four categories: (a) writers’ attitudinal/dispositional feelings (writing apprehension, attitudes to writing, and the perceived value of writing); (b) situational perceptions and operations (writing anxiety and motivational regulation of writing, respectively); (c) self-ability beliefs (writing self-efficacy and self-concept); and (d) writing learning goals (i.e., mastery or task goals versus performance ones). See Abdel Latif (2019a, 2021) for detailed discussions of conceptualization and measurement issues and the framework of writing motivation constructs.

Literature indicates that L2 students’ writing motivation is shaped by a number of factors. Collectively, these factors include: students’ personal variables (i.e., gender, age and cultural background), their writing and language performance and beliefs, and learning and instruction practices and the issues related to them such as teaching materials and practices, and teacher and peer feedback (for more details, see Abdel Latif, 2021; Karaca and Inan, 2020; Pajares et al., 2007). Instructional practices particularly play an important role in motivating students’ to write. According to Drew and Sørheim (2009), students’ motivation greatly depends on instructional practices and teacher-student relationship.

Of particular relevance to the impact of instructional practices on language learners’ motivation is the teachers’ use of motivational strategies. Teachers’ motivational strategies can be defined as the procedures used to generate, stimulate and maintain students’ learning motivation (Dörnyei, 2001; Guilloteaux and Dörnyei, 2008). Dörnyei (2001) proposed a framework encompassing the following components of motivational practices in L2 teaching: creating basic motivational conditions, generating students’ initial motivation, maintaining and protecting their motivation, and encouraging their positive retrospective self-evaluation.

Regarding the frameworks or guidelines proposed for motivating students to write, a few works have dealt with this issue. Compared to L2 learning motivational strategies, the guidelines in these frameworks are of more specific nature as they relate to writing motivation only. Besides, some of these frameworks pertain to particular writing motivation constructs rather than others. Reeves (1997), for instance, suggested the following guidelines for minimizing students’ writing apprehension: listening to fearful writers and conferring with them, varying writing modes, and preparing them for peer feedback activities. Walker (2003) also called for cultivating students’ self-efficacy through involving them in choosing writing topics, encouraging their writing self-regulation and strategy use, providing them with self-evaluation opportunities, and employing learning-oriented assessment. Meanwhile, Troia et al. (2012) viewed that students’ writing self-ability beliefs can be enhanced through fostering writing skill learnability and improveability beliefs whereas their writing learning mastery goals can be incentivized through prioritizing and modifying them and emphasizing effort attributions. Likewise, Limpo and Alves (2017) believed that teachers can improve students’ writing self-ability beliefs and mastery goals via “proposing challenging and meaningful assignments, providing frequent opportunities for success, emphasizing the process of learning, stressing self-improvement over social comparisons, giving regular progress feedback, praising for effort rather than for ability, and promoting students’ sense of autonomy” (pp. 118–119).

**Figure 1:**
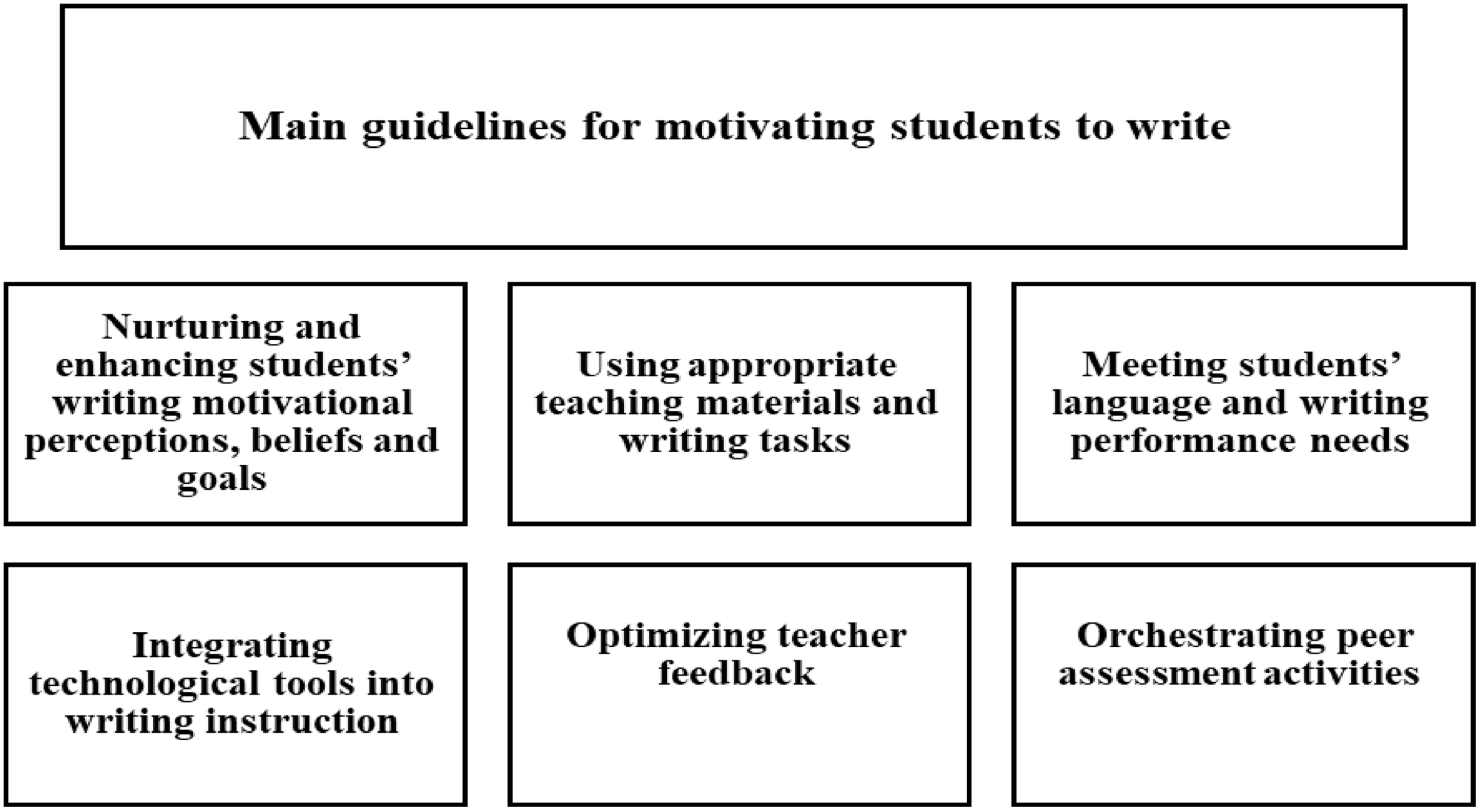
Abdel Latif’s (2021) guidelines for motivating students to write (For the list of the 42 pedagogical procedures in the six guidelines, see Abdel Latif, 2021, pp. 150–151).

On the other hand, two more detailed frameworks for fostering students’ writing motivation were provided by Bruning and Horn (2000) and Abdel Latif (2021). Bruning and Horn (2000) proposed a set of writing motivation procedures related to the following four guidelines: cultivating students’ functional beliefs about writing, engaging them in performing authentic tasks, developing a supportive learning environment, and creating a motivating learning atmosphere. More recently, Abdel Latif (2019b, 2021) suggested six main guidelines for motivating L2 students to write; each guideline has a list of pedagogical procedures, totalling 42 ones for all the six guidelines. The six guidelines are: (a) nurturing and fostering students’ writing motivational perceptions, beliefs and goals (7 pedagogical procedures); (b) using appropriate teaching materials and writing tasks (6 procedures); (c) meeting students’ language and writing performance needs (6 procedures); (d) integrating technological tools in writing instruction (6 procedures); (e) optimizing teacher feedback (9 procedures), and (f) orchestrating peer assessment activities (8 procedures). Each guidelines with its pedagogical procedures can foster particular dimensions in students’ writing motivation. These guidelines and procedures are not all used at one time but employing each depends on the stages of the writing course and students’ needs. Figure 1 shows the six motivational guidelines proposed by Abdel Latif (2021).

As noted above, some guidelines have been proposed for motivating language learners and also for motivating students to write. Chronologically, early frameworks of the two types have almost occurred in the same period (for example, Dörnyei, 2001 versus Reeves, 1997; Bruning and Horn, 2000). Contrarily, early published frameworks of language teacher motivational strategies have synchronizing been accompanied by empirical relevant studies (e.g., Dörnyei and Csizér, 1998) while research on writing teacher motivational strategies seems to have only occurred in the last decade. Since this writing motivational strategy research strand is rather recent, not many relevant studies have been published. This issue is further explained in the next section.

## 3 Previous studies

Previous studies on motivational strategies in writing classes are of three main categories: (a) interventional studies; (b) studies investigating students’ perceptions of the motivational impact of writing instruction; and (c) studies dealing with the realities of writing teachers’ use of motivational strategies. Many interventional studies of writing motivation have been published. The studies reported by Cruz Cordero et al. (2023), Zarrinabadi et al. (2023), Kim and Kim (2024), and Shen and Bai (2024) are examples of the recently published ones in this research strand. In their review of various issues in the early twenty-first century writing motivation research conducted in school settings, Camacho et al. (2021) highlighted the positive impact of some teaching treatments utilizing strategy instruction, collaborative writing, task type, and digital tools (e.g., blogs, wikis, games and web-based applications) on motivating students to write. Likewise, Abdel Latif (2021) reviewed writing studies experimenting the following six types of instructional treatments for motivating students to write: technology-supported writing instruction, writing strategy instruction, feedback provision techniques, genre-based instruction, writing task interest-based instruction, and therapeutic training. Of these instructional intervention types, technology-supported, strategy, genre, and task-interest-based instruction were specifically effective in developing students’ writing motivation.

There have also been many studies on students’ perceptions of the motivational impact of particular writing instruction types. Some of these studies revealed that students’ writing demotivation may be developed as result of inappropriate instruction practices (Atay and Kurt, 2006; Tsao et al., 2017), and the lack interesting teaching materials (Lo and Hyland, 2007). Other studies have dealt with the motivational impact of writing teacher feedback. One relevant large-scale study was reported by Yu et al. (2020) who explored how 1,190 Chinese university students perceived the impact of the following feedback types on their writing motivation: (a) scoring feedback given according to some descriptors; (b) process-oriented feedback given on multiple text drafts; (c) expressive feedback encompassing praise, criticisms and suggestions; (d) peer feedback; (e) students’ self-evaluation of their own texts; and (f) written corrective feedback. Their participant students’ writing motivation was found to be hindered by both process-oriented and written corrective feedback, but fostered by scoring, peer and self-feedback, and expressive feedback. These results emphasize the important role classroom feedback practices play in writing motivation.

Some other studies have explored writing students’ perceptions of potential teacher motivational strategies. In a qualitative study, Mali (2017) used an open-ended questionnaire and sample lesson plans to explore 65 Indonesia university students’ perceptions of teachers’ instructional practices deemed motivating for them. Mali identified 120 questionnaire statements indicating the strategies the students perceived to be motivational in their writing classes. These strategies were related to: individualized material explanation and feedback, utilizing supportive technologies, engaging students in collaborative writing tasks and peer feedback, making jokes, playing songs, creating a friendly atmosphere with students, sharing learning strategies to students, and enabling students to be autonomous in their language learning. In another learner-centred study at a Hong Kong university, Lee and Lin (2022) investigated the motivational strategies teachers use in postgraduate English academic writing courses. They collected guided reflective pieces from 59 doctoral students who were asked to reflect upon the motivational strategies their teachers used regularly, and to describe the motivational impact of such strategies on them. This study showed that the writing teachers’ effective motivational strategies as reported by their students include: using games and group work, using additional learning materials, giving students more practice, raising students’ awareness of their errors, and giving individualized instructional attention.

Only a few studies have been conducted on the realities of writing teachers’ use of motivational strategies. These studies have been concerned with different international language learning environments. Saranraj et al. (2014), for instance, examined 19 teachers’ motivational strategy use at an Indian university through using semi-structured interviews and a questionnaire with items assessing 27 instructional strategies. Their results showed that the motivational strategies the teachers reported using most frequently were raising students’ awareness of the value and importance of the target activity, modeling task performance, and describing the task properly.

Two other studies on the realities of writing teacher motivation have combined teacher and student data. In a Norwegian high school, Rosina (2017) investigated teachers’ motivational strategies in L2 writing classes through collecting questionnaire data from 100 students and conducting interview with three of their instructors. The teachers in Rosina’s study had similar motivational strategies in their writing classes, and their strategies were associated with students’ writing motivational levels. These teachers’ motivational strategies include: using videos and visual aids, supporting students’ writing through reading sources, providing students with positive and individualized feedback, and encouraging them through coursework marks. The teachers were also found unaware of more or less effective motivational strategies, and had difficulties in providing students with the needed motivational support due to time constraints. In the Singaporean higher education context, Cheung (2018) investigated the relationship between writing teachers’ motivational strategies and students’ motivation. The teachers taking part in this study were provided with a one-hour training in using motivational strategies in writing classes. The training was based on Dörnyei (2001)'s motivational strategy framework. The data was collected through surveying 344 students’ perceptions, and observing 13 teachers in writing classes and surveying their practices. The observational data in Cheung’s study showed that the strategies the teachers used pertained to generating task-specific motivation and maintaining it, and encouraging positive and retrospective self-evaluation. The results also revealed that the higher frequency of the teachers’ reported use of strategies for generating students’ initial classroom motivation was positively associated with students’ positive attitude and high self-confidence.

Some points are noteworthy about the above-reviewed scarce studies on the realities of teacher use of writing strategies. First, the recent dates of publishing or reporting these few empirical works indicate that investigating writing teacher motivational strategies is a recent research strand. In contrast to this recent and scant research, teacher motivational strategies gained much earlier attention in general language learning motivation studies (e.g., Dörnyei, 2000, 2001; Dörnyei and Csizér, 1998). Overall, the above-reviewed studies can be regarded as initial attempts in exploring L2 writing teacher use of motivational strategies. Second, these studies have investigated motivational strategies in writing classes from different research angles. Third, interviews and questionnaires are two commonly used data sources in these studies, though qualitative data is relatively more common. Moreover, in developing data sources and analyzing data, the above studies have depended on general language learning motivational strategies frameworks (e.g., Dörnyei, 2001; Guilloteaux and Dörnyei, 2008). Arguably, drawing upon the more relevant frameworks proposed for motivating students to write (e.g., Abdel Latif, 2021; Bruning and Horn, 2000) may reveal richer insights into writing teacher use and awareness of motivational strategies; an issue that seems to have been tackled only in language motivation research (e.g., Beshir, 2017; Waddington, 2018). Finally, previous studies also indicate that teacher use of motivational strategies is context-related. In other words, the use of such motivational strategies may vary from one context to another. As for the factors influencing writing teachers’ use of motivational strategies, these are yet to be explored.

Since research on writing teacher motivational strategies is still in its infancy, further studies are needed in this strand to address the above-mentioned methodological and contextual gaps. The previous few studies are not without their limitations which have been mainly caused by the general motivational frameworks used and the nature of data collected. As a result, they have revealed a limited range of motivational strategies in writing instruction. Besides, the context-specific nature of writing teacher motivational strategies calls for exploring them in different international L2 settings. Accordingly, in-depth studies in this area could have important implications for improving L2 writing instruction and promoting students’ writing motivation in specific language education environments. Their findings could also be of utmost importance to those interested in nurturing students’ writing motivation, raising writing teachers’ awareness of motivation strategies, and developing more robust survey instruments for assessing these strategies.

Taking the above-mentioned issues into account, the present study explored Saudi university EFL teachers’ perceptions of their students’ writing de-motivation and its causes and their use of motivational strategies in English writing classes. The study also investigated the potential impact of students’ writing competence and class size on teachers’ use of motivational strategies. Previous research suggests that writing competence correlates positively with students’ writing motivation (e.g., Abdel Latif, 2015, 2021; Ferris, 2002; Karlen and Compagnoni, 2017; Teng et al., 2020). Likewise, general language education literature also implies that large class size could negatively influence teachers’ ability to use motivational strategies (Cheng and Dörnyei, 2007; Dörnyei, 1998; Guilloteaux, 2013). This study seems to be the first attempt of its kind in addressing writing teacher motivational strategies in Saudi Arabia; recent writing motivation studies in this context have tackled issues other than instructional motivational strategies (e.g., Abdel Latif et al., 2024; Abdel Latif et al., 2024; Abdel Latif et al., 2024; Alsahil et al., 2024). Earlier L2 writing research in the Saudi context was mainly concerned with issues such as students’ linguistic errors, rhetorical problems and writing processes, and their responses to particular instructional techniques (for a comprehensive review, see Abdel Latif, 2011). As it seems, the realities of writing teacher motivational strategies have hardly been given any research attention in Saudi Arabia. The originality and significance of the present study stems from its context which has unique cultural and educational characteristics. Issues such as single-gender education, and the nature of English writing instruction and difficulties at Saudi universities could differently shape students’ writing de−/motivation and their teachers’ motivational strategies. Accordingly, the unique contribution of the present study lies in offering insights into writing teacher motivational strategies from the Saudi context drawing upon a more detailed and relevant framework (Abdel Latif, 2019b, 2021).

## 4 The present study: research questions and method

As implied above, the present study tried to answer the following three research questions:

1. How do Saudi university EFL teachers perceive their students’ writing de-motivation symptoms and sources?
2. How do these teachers try to motivate their students to write and participate in classroom writing activities?
3. To what extent do students’ writing competence and class size influence teachers’ use of motivational strategies?

We drew upon qualitative data to answer these research questions through using semi-structured interviews which enabled us to study the target issues from a more in-depth angle.

### 4.1 Research setting and participants

The study was conducted with a sample of faculty members who have taught writing to English majors at five Saudi universities. In the 4-year English language programmes these students attend at the five universities, English writing is taught as a core curriculum course over 4–5 terms depending on the study plan adopted by each college. In the multiple English writing courses taught, students learn writing different essay genres, including narrative, descriptive, opinion, and argumentative essays. The class size in writing courses relatively varies from one to another university, but according to the interviewees from the five universities it normally ranges from 20 to 35 students and it increases in female campuses in which a larger number of students study English as an academic major compared to male campuses.

Thirty-three faculty members took part in this study, 17 males and 16 females. They were teaching English writing at five Saudi universities (8 at University A, 7 at University B, 7 at University C, 6 at University D, and 5 at University E). In this study, we used the purposive sampling approach because we attempted to collect data from participant teachers with writing instruction experiences. We also decided to collect interview data from participants working at five universities as this would make the sample represenstive enough of writing teachers in the Saudi higher education system. An experience of teaching more than two writing courses was a pre-requisite for inviting the participants to take part in the study. Prior to starting the data collection process, the authors communicated with colleagues at the five universities to get a list of the faculty members teaching writing courses, and only those with the target writing instruction experience pre-requisite were invited through emails or phone calls to take part in the study. All the participants were PhD holders and they were of different academic ranks. The 33 teachers had varied teaching experiences ranging from three years to twenty-two years. They had also taught a number of English writing courses, ranging from 3 courses to more than 20 courses. With regard to their nationalities, the majority of the participants were Saudis (*n* = 25), and the other participants were: Egyptian (3), Jordanian (2), Sudanese (2), and Yemeni (1). All the participant teachers took part in the present study voluntarily and based on informed consent.

### 4.2 Semi-structured interviews

The present study made use of semi-structured interviews as its only data source because they allow a two-way communication mode between the researcher and interviewees, and thus help in understanding the what and why of the phenomenon investigated and allow raising follow-up questions about pertinent issues. We developed a set of guiding semi-structured interview questions in light of the research questions and the relevant literature. An expert language researcher read the guiding interview questions for face validity check, and confirmed they were appropriate for the research purpose and questions. These guiding questions focused on the teachers’ language and writing instruction experiences, their conceptualizations of students’ writing motivation, their perceptions of students’ writing de-motivation symptoms (i.e., signs) and sources, the strategies they use for motivating students to write and participate in classroom activities, the potential influence of students’ writing competence and class size upon their use of motivational strategies, and how the teachers associate students’ writing motivation in their classes with the instructional procedures, teaching materials and topics used, technology use, and teacher and peer feedback (see the guiding interview questions in Appendix 1). In developing the guiding interview questions about the teachers’ perceptions of students’ writing de-motivation symptoms and sources and their common motivational strategies (questions 2–4), we tended to raise broad questions to gain insights into the teachers’ actual writing de-motivation diagnosis practices and general de-motivation alleviating strategies. For the interview questions 5–11, we depended on reviewing literature on writing de−/motivation correlates and instructional motivational strategies.

### 4.3 Data collection and analysis

The data collection stage lasted for 6 weeks in which we obtained interview protocols from the 33 teachers who responded positively to our participation invitation. The semi-structured interviews were conducted individually with each participant. Both the second and third authors interviewed the male and female participants, respectively. The larger number of interviews (*n* = 18) were conducted on a face-to-face basis, whereas 10 participants were interviewed through online voice applications due to distance, and five other participants preferred to answer the interview questions in a written form in English. Since we were not able to raise follow-up questions in response to the written answers provided by five participants, this small interview portion has limitations in this regard; however, it added detailed information which helped us profile the teachers’ writing de-motivation diagnosis and motivational strategies. In the face-to-face and online interviews, each participant teacher was interviewed in English or Arabic, or using a mixture of both languages, depending on their language preference. The interviews were guided by the questions we developed, and follow-up questions were also raised for eliciting the interviewees’ pertinent opinions and narratives. All interviews lasted for 55–70 min.

The data analysis started with transcribing the interviews conducted in English, and translating the Arabic interviews and interview parts and then transcribing them in English. The translations of the Arabic interview parts were made by the second and third authors, and reviewed and edited by the first author for meaning preservation verification; all the three authors are Arabic-native speakers with previous Arabic-English-Arabic translation experiences. Thus, we had all the 33 interviews transcribed in English. In our interview data analysis, we depended on the following procedures: exploring the data and initially categorizing it, identifying the descriptions related to each category in the interview protocols, reviewing and refining and initially identified categories, and confirming the evidence emerging from the data (Lodico et al., 2006). We read the interview protocols independently and categorized the emerging themes in them deductively using our research questions as broad guidelines and inductively through examining the sub-themes in each main category. Following this individual analysis, we met online to discuss the emerging themes each one of us identified. Through our online group discussion of the emerging themes in the individual analyses, we further revised and reconfigured them (Merriam, 1998), resolved the discrepancies in the data analysis, and reached agreed-upon labels for all the sub-themes. The trustworthiness of our interview data analysis was verified by an expert researcher who read four analyzed interview protocols to determine how much he would agree with the analysis made. The collaborator researcher had a very high agreement with the themes and categories we identified (93%) in the four protocols, and his comments were taken into account for refining some few dimensions in the data analysis. Guided by Abdel Latif (2021)'s writing motivation framework and motivational strategy guidelines, we organized these into categories related to the research questions.

## 5 Results of the study

In the following sub-sections, we present the results of the data analysis in light of the research questions. Each sub-section includes the answer of one research question.

### 5.1 The teachers’ perceptions of students’ writing de-motivation symptoms and sources

The teachers’ interview answers showed they had varied conceptualizations of writing de-motivation symptoms or signs. Collectively, the 33 teachers identified the following seven symptoms of their students’ writing de-motivation: procrastinating or skipping essay assignment submission, having a little engagement in classroom writing activities, showing a negative attitude toward writing, copying online materials or others’ writing, skipping writing classes, having a low-perceived value of writing, and experiencing writing block. Table 1 provides the frequencies of these writing de-motivation symptoms as reported by the teachers, along with sample interview excerpts indicating them.

**Table 1:** The teachers’ conceptualizations of writing de-motivation symptoms.

The writing de-motivation symptom	No. of interviewees referring to it	Sample interview excerpt
Procrastinating or skipping essay assignment submissions	12	*I usually notice students’ inadequate writing motivation when they ask me to extend the deadline or when they do not submit required essays.* (Teacher 31)
Having a little engagement in classroom activities	11	*When these students participate in writing activities or discussions, they pretend to do brainstorming when I walk past that group but in reality you can tell they are zoned out.* (Teacher 20)
Showing a negative attitude toward writing and its assignments.	8	*I typically notice low writing motivation in my writing classes when students consistently exhibit a lack of enthusiasm towards writing assignments… These students sometimes openly say they do not like writing or show signs of reluctance to start writing assignments.* (Teacher 24)
Copying online materials or others’ writing	6	*They are the students who rely on looking at their classmates’ writing and copying it. Sometimes they may also copy online essays or ask others to write essays for them. … I can easily notice this in the essays they submit as there is a wide difference between the students’ low levels and the high quality of the essays they submit.* (Teacher 1)
Skipping writing classes	5	*The students who are not motivated skip writing classes and do not attend them regularly.* (Teacher 7)
Having a low-perceived value of writing	4	*Some students perceive little relevance of writing skills in real life…This leads them to take writing courses only because they are mandatory, and not because they expect to benefit from them.* (Teacher 19)
Experiencing writing block	1	*They feel that writing is difficult and they do not know how to start the task, and so they have no aptitude to write in English.* (Teacher 5)

The frequencies of the symptoms imply how common the teachers have found them in their writing classes. As may be concluded, some teachers mentioned one symptom of students’ writing de-motivation, while others referred to two or more. For example, the teacher in the following interview excerpt is talking about several signs such as experiencing a negative attitude toward writing, skipping writing classes, and having little engagement in classroom activities:

*Based on my teaching experience, I often identify students with a lack of writing motivation through certain behaviours. … Occasionally they do not attend course classes, and they have a lack of interest in learning writing. … They show a limited participation in topic discussions or group activities. … And they also have frequent distractions or off-task behaviours during classroom activities.* (Teacher 14)

Regardless of the number of de-motivation signs mentioned by each teacher, all the symptoms they gave mainly pertain to the attitudinal dimension of writing de-motivation, which includes students’ negative attitudes toward writing, avoidance behaviors, and the perceived value of writing. In their description of the de-motivation sources, the teachers also talked about students’ low language and writing ability beliefs, but they did not refer to any other signs or symptoms related to students’ situational experiences (e.g., writing anxiety and low self-regulation), or the lack of writing achievement goals. Such limited conceptualization seems to have also negatively influenced the variety of motivational strategies the teachers use in their writing classes; this issue is discussed in the following subsection.

On the other hand, the interviews revealed important issues about the teachers’ perceptions of their students’ writing de-motivation sources. Overall, the 33 teachers identified five causes; these are: students’ poor linguistic knowledge and writing ability, uninteresting writing topics, ineffective teaching materials and techniques, previous poor writing learning experiences, and the cognitive nature of writing tasks. Table 2 gives the frequencies of the teachers’ mentions of these causes or sources and sample interview parts. Such frequencies suggest how often they occur in Saudi university writing classes.

**Table 2:** The teachers’ perceptions of their students’ writing de-motivation sources.

The source of writing de-motivation	No. of interviewees referring to it	Sample interview excerpt
Poor linguistic knowledge and writing ability	19	*There could be various factors causing students’ lack of motivation to write in English, but I think the main factor is their poor English knowledge or their poor level in English essay writing, particularly in vocabulary. … Many students usually avoid participating in classroom activities in order not to be criticized for their language level.* (Teacher 1)
Inappropriate writing topics	8	*Students lose motivation when they feel the writing topic is irrelevant or not interesting. … They will not participate in the writing activities because they have no idea about the topic…. I also notice this case of losing motivation when students feel the topic is more difficult than expected; I mean it does not match their academic level.* (Teacher 29)
Ineffective teaching materials and techniques	7	*If textbooks are boring or teaching is monotonous, it will be difficult for students to stay motivated. … Lack of writing motivation can be also caused by any brief feedback students get from their writing teachers. … Students may struggle immensely if they have unclear feedback.* (Teacher 10)
Previous poor writing learning experiences	4	*Another cause of students’ negative writing motivation is their poor level in writing during the pre-university stage… They did not learn how to write good English texts in schools.* (Teacher 14)
Cognitive nature of writing tasks	2	*Some students feel that writing in general is difficult, and unlike practicing other language skills, they feel writing takes a long time.* (Teacher 32)

On their descriptions of the role played by linguistic knowledge and writing ability levels, the interviewees (*n* = 19) indicated it is strongly associated with their students’ writing motivation; as one interviewee explained:

*Students with a good writing performance are always motivated to write. … But students with a poor performance are usually less motivated. It is rare to find a high-level student low-motivated. If this happens, it is usually because the writing topic is less challenging and mediocre.* (Teacher 23)

The majority of these 19 interviewees linked low-motivated students’ poor writing with their inability to use appropriate vocabulary and grammar in their writing. Some other interviewees associated it with their inability to generate ideas and organize them even in their L1 (i.e., Arabic). For both teams of teachers, students’ low-perceived level of English language proficiency and writing ability causes them not to participate actively in classroom writing activities because they fear to be criticized for their poor texts.

As for the influence of writing topics on students’ motivation, the eight teachers highlighting this issue generally believed that a writing topic can be motivating to students if it is interesting, familiar to them, challenging enough and matches their levels. One interviewee also mentioned that topic interest can be a gender-related issue:

*Female and male students may also react to some topics differently; for example, if female students are asked to write about sports or social issues, they will find this discouraging.* (Teacher 18)

The seven teachers referring to the use of ineffective teaching materials and techniques as a potential cause of writing de-motivation thought that for writing learning materials and instruction to be motivating, they should not be boring, very difficult and should match students’ interests. Meanwhile, these teachers had varied views regarding the perceived motivational impact of the writing teaching materials they use in their classes. Some teachers believed these teaching materials were motivating enough to students while others though they did not meet students’ needs. Finally, a fewer number of teachers attributed students’ writing de-motivation to learning previous experiences and the cognitive nature of writing tasks (*n* = 4 and 2, respectively). While the first cause suggests students’ long-term negative writing learning experiences have had a de-motivational impact on them, the second cause implies the cognitively demanding nature of the text composing process does not match some students’ learning styles.

### 5.2 The teachers’ instructional motivational strategies

The interviewed teachers reported using eight main strategies for motivating their students to write and to participate in classroom activities. Table 3 shows these strategies, the number of the interviewed teachers referring to them, and related sample interview excerpts. The motivational strategies the teachers reported using are: optimizing teacher feedback, considering and negotiating writing topic choice, engaging students in collaborative writing and assessment activities, getting them to use technological tools in writing learning, adapting teaching materials, cultivating students’ writing motivational beliefs, incentivizing their participation in classroom activities, and relieving students’ concerns about making errors. The reported frequencies of these motivational strategies indicate that some of them are more commonly used than others. The motivational strategy with the highest frequency is optimizing feedback (*n* = 22 teachers). According to the 22 teachers, they tried to optimize their feedback through different strategies, including: providing students with constructive and timely feedback, using individual feedback more than group (i.e., whole class) feedback, varying feedback content and focus, alleviating criticism in individual feedback, and referring to texts anonymously in group feedback.

**Table 3:** The teachers’ reported motivational strategies.

The motivational strategy	No. of interviewees referring to it	Sample interview excerpt
Optimizing teacher feedback	22	*I try to foster a positive feedback culture to avoid students’ sensitivity to criticism in essay comments. … I tend to write a positive comment before the negative one, and change the areas of writing I praise. … I also explain the reason for any criticism and say it does not reflect the students’ level. … If comments are given to the whole class, they are always without names.* (Teacher 11)
Considering and negotiating writing topic choice	17	*I try to choose the writing topic students like or have ideas about. … Sometimes, I allow students to choose the writing topic. … In the classroom, I also get them to perform writing task in separate stages. I mean they first do planning stage, and then the writing and revising stages.* (Teacher 24)
Engaging students in collaborative writing and assessment activities	13	*I usually use pair or group work writing tasks to create a cooperative learning environment so that students feel motivated and motivate each other, and alleviate anxiety for everyone. … I believe encouraging students to participate in writing activities is the way to go.* (Teacher 23)
Getting students to use technological tools in writing learning	12	*I use apps like Grammarly in classrooms to help students notice their errors. But for lower-level students, I use Nearpod or Google Docs to encourage collaborative writing and discussion.* (Teacher 2)
Adapting teaching materials and techniques	10	*Even though we adhere to the prescribed textbook, I use additional and more engaging supplementary materials. … I try to diversify the materials and use extra materials to enhance students’ understanding and motivation.* (Teacher 15)
Cultivating students’ writing motivational beliefs	9	Teacher 33: *At the very beginning of each writing course, I always try to show them the importance of writing to their future career and to proficiency in English. … I explain that writing holds substantial relevance in real-world contexts.*
Incentivizing students’ classroom participation	8	*I make efforts to help students with low writing de-motivation become more motivated in my classes. I assign marks for participation.* (Teacher 1)
Relieving students’ concerns about making errors	3	*I try to push students to be motivated in their writing and not to worry about errors. … I explain they are not held accountable for grammatical or stylistic errors. I always say to them: the more mistakes you make, the better writer you become.* (Teacher 9)

With regard to the issue of considering and negotiating writing topic choice, the 17 teachers reporting using this motivational strategy said they select the topics matching students’ interests and appropriate to their background knowledge, assign students easy writing tasks, engage them in choosing the topics they want to write about, or getting students to perform the one task as separate sub-tasks (i.e., planning, writing and revising). In the following interview, a female teacher is referring to one of these approaches in writing topic selection:

*When students find topics irrelevant or not interesting, I try my best to personalize writing topic as per their interest. … For example, if a writing unit discusses sports, I would make the task to be about a sport they like or wish to do it in real life … This sort of personalizing writing tasks really encourages students to write.* (Teacher 16)

Thirteen teachers mentioned engaging students in collaborative activities as a way for motivating them to write. Seven out of these 13 teachers mentioned using collaborative activities in the form of pair or group work writing tasks, while the other six teachers reported using peer assessment activities. It is noteworthy that the majority of the interviewees (*n* = 27) had a negative attitude toward using peer assessment. According to these teachers, peer assessment could have detrimental effects on students with low writing competence, sensitivity to peer criticism, or lack of seriousness. The following three teachers elaborated on this point as follows:

*I do not prefer getting students to evaluate the work of their classmates. Students may feel very sensitive and embarrassed. …. Generally and typically, students exhibit reluctance in evaluating their peers’ essays.* (Teacher 3)

*Unfortunately, my students’ levels are not that good…. A few students can add to their classmates. … So, I prefer to avoid getting them to correct their peers’ errors.* (Teacher 19)

*I used to do peer evaluation several years ago, but I noticed that most students deliberately write nice comments and do not point out errors.* (Teacher 21)

The narratives of the six teachers who reported using peer assessment activities indicate they implement them non-regularly and cautiously. Two of these teachers said they use these activities a few times a term, while the other four teachers said they use them conditionally, for instance after students know each other very well, or when they have a suitable writing competence level. Three teachers also said that preparing students for these activities is another complicated issue. The following interview excerpts show these cases:

*I use peer assessment just to break the ice, and help students notice their errors.… But I use it only four weeks after the beginning of the writing course because peer revision is resisted when learners do not know each other. … As time passes, students’ shyness decreases. … It always helps to use a rubric for these tasks.* (Teacher 7)

*I use peer evaluation if students in my class are of intermediate and higher levels only. In pre-intermediate classes, peer assessment normally causes much embarrassment for low-level students. … Before getting students to participate in peer evaluation activities, I often establish clear expectations and guidelines and create a supportive and respectful classroom environment in order to overcome resistance. … I then observe the interaction of students in each group to make sure everyone participates. I also assist students when necessary.* (Teacher 16)

*I help students have a positive peer evaluation experience by addressing their concerns, and by fostering a culture of respect and constructive criticism. … I start with talking to students individually to find if they resist participating in peer evaluation activities. Once I understand their concerns, I can deal with them. I also try to help them understand the benefits of peer evaluation. … To help students have a guided peer assessment activity, I provide guidance on giving constructive feedback, and monitor their feedback participation.* (Teacher 20)

The above interview excerpts suggest that implementing peer assessment in L2 writing courses requires some particular conditions leading to the desired motivational impact.

As for the role of technology in motivating students to write, the interviewed teachers were divided about this issue. The larger group of the teachers reported a negative attitude toward using technological tools in writing classes. For these teachers, using technological tools in writing classes is not beneficial and can cause unfavorable outcomes:

*I believe technology affects students negatively. So, I do not allow them to use it in the classroom because if students were introduced to particular applications, essay plagiarism and auto-correction will increase. … Students will learn writing better without technology.* (Teacher 11)

*I do not feel technology is important in writing classes. It’s better to give the student the opportunity to try, make mistakes, and find the solution. But I only encourage my students to use grammar and spelling checking tools at home, and they usually like them.* (Teacher 28)

The 12 teachers who mentioned making use of technology in writing classes to motivate students referred to using tools such as Grammarly, Nearpod or Google Nearpod, Google Docs or blogs. Overall, these teachers’ answers indicate they do not make great use of technology in their writing instruction. Like their attitude toward technology use, the larger number of the teachers did not feel a dire need for adapting teaching materials for fostering students’ motivation. These teachers viewed that students rarely get dissatisfied with the writing teaching materials used. Additionally, eight out of the 10 other teachers narrating making some kind of language teaching material adaptation said that it is only contingent upon noting dissatisfaction and de-motivation symptoms.

Only nine teachers talked about cultivating students’ writing motivational beliefs. Their narratives showed they care about some of these motivational beliefs rather than others. Specifically, the nine teachers mainly referred to their attempts to cultivate students’ motivation through highlighting the value of writing to their academic life and future careers, and, to a less extent, trying to alleviate students’ negative writing attitude. Apart from this, no teacher talked about cultivating other motivation dimensions such as helping students regulate their emotions while writing, promoting their writing self-ability beliefs, or supporting them in setting achievable goals in writing courses. Eight teachers reported trying to motivate students by incentivizing their participation in classroom activities. These teachers’ common strategy was to make a part of students’ coursework marks dependent upon their active participation in such activities. Likewise, three teachers mentioned they try to encourage students to write by relieving their concerns about making written errors. For these teachers, relieving students’ fears of error criticism could be one way for encouraging them to write.

Finally, it is worth noting that three teachers reported they do not feel obliged to care about students’ writing motivation. In other words, these teachers believed that such motivating task is not a main part of their instructional roles. In the following interview excerpts, two of them obviously stated they pay little attention to considering students’ motivation in their classes:

*The only way I tried in motivating students is choosing a topic that could match their interest or getting them to choose a topic they are interested in.* (Teacher 4)

*I do not consider students’ opinions about my criticism or praise worthy of listening to. I only provide students with detailed written and corrective feedback.* (Teacher 26)

Additionally, the interviews indicate the little attention the teachers pay to directly cultivating students’ writing motivation. When asked about this issue, one female teacher, for instance, commented, “*I do not really care about it if students remain de-motivated*” (Teacher 7).

The strategies the teachers reported using do not reflect a wide range of writing motivation procedures when compared to the previous relevant taxonomies (e.g., Abdel Latif, 2021; Bruning and Horn, 2000), see section 2. For example, the teachers’ reported motivational strategies do not have much in common with Bruning and Horn’s (2000) guidelines for cultivating students’ functional beliefs about writing, and creating a supportive learning atmosphere. These strategies do not either adequately cover Abdel Latif’s (2021) proposed guidelines for meeting students’ language needs, orchestrating peer assessment activities and nurturing students’ writing motivational perceptions. The finding that some teachers in the Saudi university context are not interested in motivating their students may partially account for the noted limited conceptualizations of writing de-motivation (see 5.1). Therefore, more attention should be paid to raising teachers’ awareness of the value of and effective ways for promoting students’ writing motivation.

### 5.3 Students’ writing competence and classroom size as potential correlates of the teachers’ use of motivational strategies

The teachers’ interview answers also helped in understanding their view on the role of students’ writing competence and classroom size as potential factors influencing their use of motivational strategies in L2 writing classes. Regarding the role of students’ writing competence, most teachers (*n* = 24) said it does not greatly influence their use of varied motivational strategies. For some of these teachers, competent student writers are normally motivated and therefore they only motivate low-level students who are de-motivated as a result of writing skill deficiencies. For another team of these teachers, motivational strategies remain unchanged when dealing with both high- and low-level writers.

Conversely, only nine teachers mentioned varying strategies for motivating students with different writing levels. The following two cases are typical of the narratives reported by the teachers in this group:

*Good students usually struggle with uninteresting topics. … I try to make the topics personal and ask them to search for background information to get their ideas flow. … Poor students struggle with writing mechanics and basics and feel overloaded easily. So, I try to ask them to use Grammarly to know their errors and edit their writing instead of feeling stressed about everything.* (Teacher 12)

*Students with good performance are always motivated. Many writing activities and tasks in the course are too easy for them. So, what they need is to work on more difficult tasks. For example, I get them to do more challenging writing activities during classes, leaving easier activities to poor students as they will feel more motivated when answering them correctly.* (Teacher 30)

Collectively, the nine teachers mentioned motivating high-level students through changing writing topics and getting them to do more challenging writing activities or tasks, and they reported motivating low-level students through getting them to do easier tasks, supporting their learning, or guiding them to digital resources for completing their essays or revising them.

Regarding class size, all the teachers congruently viewed that it is a more influential factor in their use of writing motivation strategies. According to them, the fewer number of students are easier to motivate and to pay individualized attention to; for example:

*Of course it is better to have a fewer students so that you have the time to focus on each student. So, a smaller group tends to be more manageable. In larger classes, it may be more challenging to provide individualized attention to make effective use of strategies such as peer collaboration and using technology for personalized feedback. In smaller classes, more individualized motivational support and one-on-one feedback can be provided.* (Teacher 8)

*Certainly, the number of students in the course can influence the solutions for related problems. In larger classes, group activities and collaborative writing can encourage participation and engagement. In smaller classes, more attention, individualized feedback and tailored instruction can be provided to deal with specific challenges related to writing motivation.* (Teacher 19)

The above two interview parts suggest that even if the teachers are willing to exert considerable efforts in fostering students’ writing motivation, the large number of students in one class can hinder their task. That is why minimizing students’ numbers in English writing classes is key to helping the teachers in accomplishing their motivational task.

## 6 Discussion and conclusions

The present study aimed at exploring Saudi university EFL teachers’ diagnosis of students’ writing de-motivation symptoms (i.e., signs or indicators) and causes, and the strategies they use to motivate students to write and participate actively in classroom activities, i.e., their motivational strategies. Overall, we did not note tangible differences among the teacher groups at the five universities in these three dimensions; they were generally similar in their diagnosis of writing de-motivation indicators and causes, and also in their reported motivational strategies. The study uncovered important results about the teachers’ writing motivation literacy and the efforts they allocate to get their student writers motivated. Compared to the previous few studies (Cheung, 2018; Lee and Lin, 2022; Mali, 2017; Rosina, 2017; Saranraj et al., 2014), using semi-structured interviews and framing data collection and analysis based on a more relevant framework (Abdel Latif, 2021) have helped in revealing different insights into the realities of motivational strategies in L2 writing classes.

With regard to the teachers’ conceptualizations of writing de-motivation, these were found to be rather limited as it turned out they lack a comprehensive awareness level of what it involves. Obviously, the teachers’ conceptualizations fall only in the attitudinal and ability belief dimensions of writing de-motivation (see Abdel Latif, 2021). In other words, the de-motivation symptoms mentioned by the teachers concern students’ negative attitudes toward writing, and avoidance behaviors, task procrastination, and writing block. The teachers also referred to poor linguistic knowledge and writing ability – an ability belief dimension – as a cause of students’ de-motivation. Apart from attitudes and ability beliefs, the teachers mentioned no other ones related to the situational or goal orientation dimensions of students’ de-motivation. While it is well-acknowledged that these signs may vary from one student and/or context to another, de-motivated writers normally experience attitudinal, situational, ability belief, and goal orientation symptoms – see Abdel Latif’s (2021) definition in section 2. Thus, writing de-motivation is not merely an attitudinal-ability belief construct but it is a four-dimensional one. The teachers’ limited conceptualization of writing de−/motivation could have resulted from a lack of awareness of its multiple aspects. It may have also been associated with the little attention some teachers paid to cultivating students’ writing motivation (as indicated in subsection 5.2). The teachers’ diagnosis of writing de-motivation causes is generally consistent with pertinent literature and previous research findings (e.g., Abdel Latif, 2021; Karaca and Inan, 2020; Pajares et al., 2007). It seems that writing de-motivation sources/causes occur consistently across different international educational settings.

Compared to previous research findings (e.g., Cheung, 2018; Lee and Lin, 2022; Rosina, 2017), the present study revealed a wider range of teacher motivational strategies in Saudi university English writing classes. However, the strategies the teachers mentioned using relate only to some dimensions in the motivational strategy frameworks proposed by Bruning and Horn (2000) and Abdel Latif (2021). Specifically, the teachers’ motivational strategies align adequately with the proposed guidelines of using appropriate teaching materials and writing tasks, and optimizing teacher feedback, but they align partially with the guidelines of developing a positive learning environment, cultivating students’ writing motivational perceptions and creating a motivating learning atmosphere via using technological tools in instruction. Meeting students’ language and writing performance needs and orchestrating peer assessment activities seem to be almost neglected as the teachers mentioned a very few motivational strategies pertaining to these two guidelines. Consistent with previous language motivation research findings (e.g., Cheng and Dörnyei, 2007; Dörnyei, 1998; Guilloteaux, 2013), the present results indicate that teachers are likely to be unable to use motivational strategies effectively in writing classes with a large number of students. Meanwhile, the study has not provided conclusive evidence for the interaction between teachers’ use of motivational strategies and students’ writing competence levels.

In light of the present results, it is concluded that Saudi university teachers’ motivational practices in English writing classes are yet to be enhanced. Such enhancement requires three steps. First, teachers’ writing motivation literacy or awareness needs be fostered to help them understand writing motivation dimensions and know how to motivate students properly. Second, there is also a need for activating the use of motivational strategies in writing classes. Both steps could be accomplished through teacher education programmes and in-service training workshops. Besides, teacher educators and textbook writers have the potential to play an important role in drawing teachers’ attention to relevant issues. For example, pre-service and in-service teacher educators may pay due attention to raising teachers’ awareness of writing de-motivation symptoms and causes, and to fostering their consciousness of how to help de-motivated students. Teaching supervisors could also draw teachers’ attention to some guidelines for optimizing their practices to meet students’ writing motivational needs. In addition, teacher guides could include some instructional scenarios related to diagnosing students’ writing de-motivation symptoms and alleviating them. Congruent with the present results emphasizing the central role of class size in enabling teachers’ use of motivational strategies, there is also a need for minimizing the number of students in English writing classes at Saudi universities. With appropriate writing class size, teachers’ task in getting students motivated will be easier.

The scant research on writing teachers’ motivational strategies calls for addressing multiple dimensions in this area. The realities of motivational strategy use need to be investigated in different international L2 writing learning settings. It is also important to profile a larger number of factors potentially influencing writing teachers’ use of motivational strategies, including students’ gender, educational stage and cultural context. Another issue worth investigating in future studies is how teachers’ writing motivation literacy may impact the ways they motivate students in writing classes. Future relevant studies can also draw upon other data sources such as classroom observation or combine it with semi-structured interviews or questionnaires. Such future research could help in disseminating a more effective motivation culture in L2 writing learning environments.

## Data Availability

The raw data supporting the conclusions of this article will be made available by the authors, without undue reservation.

## Appendix 1

### Guiding interview questions

1. I would to like start this interview by getting some information about your language and writing teaching experiences. Could you please tell me about your writing instruction and the writing courses ad students you have taught?
2. In each writing class, there are normally some students who do not feel motivated to write or do not like to participate in classroom activities. When and how do you usually notice students with inadequate writing motivation? Please explain in detail the behaviors of such students.
3. In your opinion, what have caused these students not to not feel motivated to write in English? Please explain in detail any potential related causes you have noted in your English writing classes.
4. Do you usually try to help students with low writing motivation in your classes become more motivated? If so, please explain in detail the things you do or the strategies you use for this purpose.
5. To what extent do students in your English writing classes feel happy or satisfied with the textbooks or instructional techniques you use? If they are not happy with them, how do you deal with this problem? Please explain in detail.
6. How many essay topics do you normally assign your students each term in your writing courses? What are the students’ reactions to the number and types of the essay topics assigned? How do you try to overcome any problems related to the students’ reactions to the number of essay assignments or essay topics? Please explain in detail.
7. Some students with good or poor writing performance alike may have a lack of writing motivation. Have you noted this case in your English writing classes? Generally, how do you try to help each type of students to become motivated? Please explain in detail.
8. Do you make use of technology for fostering your students’ motivation to write and to get them engaged in English writing classroom activities? If so, which technological aids or tools do you use in your English writing classes? And what are students’ reactions to them? Please explain in detail.
9. When commenting on students’ English essays, do some of them feel annoyed with your criticism or even bored with your repeated praise? Is this the same when giving oral or written, to an individual student or a group of students- or to students with different performance improvement levels? How do you usually try to consider students’ potential sensitivity to or boredom with your comments on their essays? Please narrate your related experiences.
10. In your English writing classes, do you get students to evaluate their classmates’ essays? In case you have noticed some students resist participating in this type of evaluation activities, how do you usually try to help these students have a positive peer evaluation experience? Please explain your related experiences in detail.
11. When dealing with all the above mentioned issues in your writing classes, do your solutions of any potential writing motivation-related problems differ depending on:

- The number of students in the course.
- The students’ academic level or progress.

Please explain in detail.
